# Total Flavonoids in *Caragana* (TFC) Promotes Angiogenesis and Enhances Cerebral Perfusion in a Rat Model of Ischemic Stroke

**DOI:** 10.3389/fnins.2018.00635

**Published:** 2018-09-12

**Authors:** Qiansong He, Shirong Li, Lailai Li, Feiran Hu, Ning Weng, Xiaodi Fan, Shixiang Kuang

**Affiliations:** ^1^Guiyang College of Traditional Chinese Medicine, Guiyang, China; ^2^Department of Neurology, Guizhou Provincial People’s Hospital, Guiyang, China; ^3^Department of Experimental Research Center, Xiyuan Hospital of China Academy of Chinese Medical Sciences, Beijing, China

**Keywords:** TFC, tMCAO, angiogenesis, neurological function, infarct volumes

## Abstract

Previous studies have demonstrated that total flavonoid extracts from *Caragana sinica* (TFC) exert multiple therapeutic effects, promote blood flow, and exhibit anti-inflammatory and antioxidant properties. The present study aimed to investigate whether TFC promotes angiogenesis and exerts neuroprotective effects in a rat model of transient middle cerebral artery occlusion (tMCAO). Male Wistar rats were subjected to tMCAO for 1.5 h, followed by 24 h of reperfusion. TFC (15, 30, 60 mg/kg) was administered for 14 days. Evaluations of neurological function were performed following reperfusion, and infarct volumes were assessed in brain slices stained with 2,3,5-triphenyltetrazolium chloride (TTC). Our results indicated that TFC significantly attenuated cerebral infarct volume and neurological deficits following tMCAO. Laser Doppler, micro-PET/CT, and MRI analyses further demonstrated that TFC reduced infarct volume and enhanced cerebral blood flow in a dose-dependent manner, with the most significant effects occurring at a concentration of 60 mg/kg. Significant up-regulation of CD31, VEGF, Ang-1, HIF-1α, delta-like 4 (Dll4), and Notch1 expression was also observed in the experimental groups, relative to that in the vehicle group. In summary, the results of the present study indicate that TFC (15, 30, 60 mg/kg) attenuates neurological deficits, reduces infarct volume, and promotes angiogenesis following MCAO in a concentration-dependent manner, likely via increases in the expression of CD31, VEGF, Ang-1, HIF-1α, Dll4, and Notch1. Further studies are required to determine the clinical usefulness and potential mechanisms of TFC in patients with cerebral focal ischemic stroke.

## Introduction

Stroke is the second leading cause of death worldwide. As most cases of stroke occur due to ischemia, the need for more effective treatment strategies for ischemic stroke remains urgent (Bacigaluppi et al., 2008). In the ischemic brain, blood supply is severely reduced in the affected areas, eventually leading to cell death/apoptosis due to a lack of oxygen and nutrients (Plate, 1999). Accumulating evidence demonstrates that ischemic brain injury can be attenuated by restoring cerebral blood flow and rescuing dying neurons (Matsumoto et al., 1990; Zhang et al., 2002; Hoang et al., 2009). Indeed, thrombolytic and neuroprotective therapies represent the two primary measures currently used to treat acute cerebral infarction. Recent research has focused on the development of agents that induce angiogenesis and exert neuroprotective effects in an effort to cure ischemic stroke. Natural compounds such as medicinal herbs, which are associated with fewer adverse effects than standard medications, may allow for safe and effective induction of angiogenesis and neuroprotection (Pierre et al., 1999).

**FIGURE 1:**
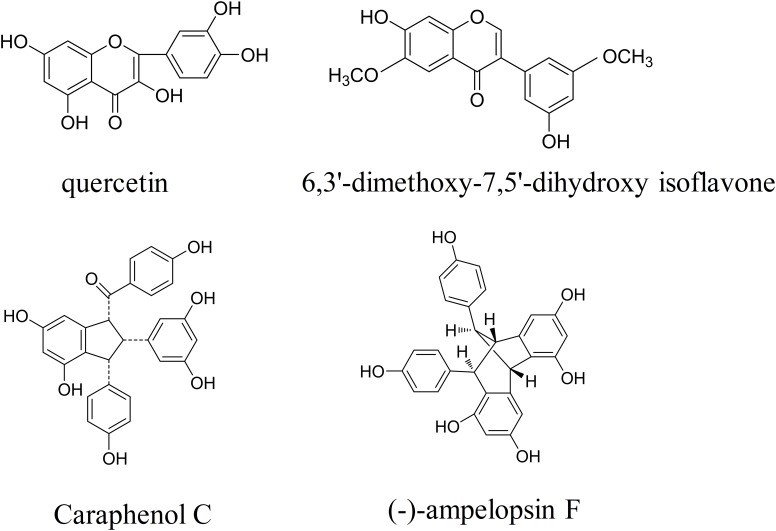
The chemical structures of four *Caragana* extract components, including quercetin, 6,3′-dimethoxy-7,5′-dihydroxy isoflavone, caraphenol C, and (-)-ampelopsin F.

*Caragana sinica* (Fabaceae), commonly known as Chinese peashrub, is widely distributed throughout China, particularly in Mongolia and Tibet. Since the 10th century, *Caragana sinica* has been used in the treatment of a variety of symptoms (e.g., colds, strains, fatigue, wheezing) (Jia et al., 1997). Accumulating evidence has demonstrated that *Caragana sinica* improves blood profiles, facilitates blood flow, clears lung-heat, promotes kidney and spleen function, and aids in the healing of bruises/contusions (Jiangsu New Medical College, 1986). In our previous chemical constituent analysis, we revealed that flavonoids contained within ethyl acetate extracts of the *Caragana sinica* root exhibit multifaceted bioactivity (He et al., 2017). Previous studies have reported that flavonoids—which are defined as polyphenols that exert cytoprotective effects—exhibit anti-inflammatory, anticancer, antioxidative, antiviral, and antibacterial properties (Wang et al., 2010, 2014; Al-Nakkash et al., 2012; Li et al., 2012; Lim et al., 2013; Lin et al., 2015; He et al., 2017). Recently, we have investigated the effects of flavonoids and several known compounds derived from *Caragana sinica* [total flavonoids in caragana (TFC)], including quercetin, 6,3′-dimethoxy-7,5′-dihydroxy isoflavone, caraphenol C, and (-)-ampelopsin F (**Figure 1**) on ischemic brain injury. In particular, we aimed to determine whether and how TFC enhances angiogenesis in rodent models of stroke.

As previously mentioned, blood supply to the affected areas is severely reduced in the ischemic brain. Previous studies have demonstrated that angiogenesis is critical for remodeling of the neurovascular matrix in regions affected by stroke and other neurodegenerative diseases, contributing to the generation of functional nerves and synapses (Beck and Plate, 2009; Hermann and Zechariah, 2009; Yang et al., 2013). Additional studies have indicated that angiogenesis can improve the perfusion of ischemic brain tissue (Plate, 1999; Lin et al., 2002), and that the degree of angiogenesis is associated with survival rates among patients with stroke (Krupinski et al., 1994). Indeed, higher vascular density has been associated with increased survival times following ischemic stroke (Alonso de Leciñana et al., 2014). Furthermore, accumulating evidence suggests that angiogenesis improves neurological function and reduces cerebral infarction volumes in rodents (Chen et al., 2014; Meng et al., 2014; Yang et al., 2015).

Vessel formation and maturation during angiogenesis are primarily influenced by hypoxia inducible factor-1α (HIF-1α), angiopoietin-1 (Ang-1), and vascular endothelial growth factor (VEGF). It has been collectively suggested that HIF-1αplayed a significant role in cerebral angiogenesis and showed neuroprotective effect in ischemic stroke (Doeppner et al., 2016). HIF-1α is considered as a main regulator in the process of hypoxia or ischemia, and HIF-1α can mediate the VEGF/Notch1 signaling pathway in the development of collateral circulation (Li et al., 2014). Previous studies have indicated that VEGF increases microvascular permeability and is essential for proper functioning of the embryonic vascular system (Dvorak et al., 1995; Yancopoulos et al., 2000; Shi et al., 2016). In addition, VEGF signaling can enhanced members of the Delta-like/Jagged/Notch-family, proteins notably important in angiogenesis. Delta-like 4 (Dll4) is an arterial endothelial specific ligand for Notch1 receptor, and both of them are more specifically involved in angiogenesis (Gale et al., 2004; Hainaud et al., 2006; Kume, 2012). Ang-1 plays a critical role in physiological and pathological angiogenesis during embryonic and postnatal life, and in the mutation of freshly formed blood vessels (Carmeliet and Collen, 1997; Thurston et al., 2000; Brindle et al., 2006; Al Sabti, 2007). Moreover, Ang-1 is essential for vascular maturation and stabilization via endothelial attachment to extracellular matrices (Thurston et al., 2000). Several studies have suggested that VEGF cooperates with Ang-1 and other factors: HIF-1α, Dll4, and Notch1 to promote vascular formation (Dvorak et al., 1995; Carmeliet and Collen, 1997; Thurston et al., 2000; Yancopoulos et al., 2000; Gale et al., 2004; Brindle et al., 2006; Hainaud et al., 2006; Al Sabti, 2007; Kume, 2012; Chen et al., 2014; Li et al., 2014; Meng et al., 2014; Doeppner et al., 2016; Shi et al., 2016, 2018). Therefore, in the present study, we investigated whether TFC influences angiogenesis by enhancing the expression of VEGF, Ang-1, HIF-1α, Dll4, and Notch1 and whether treatment with TFC can alter long-term functional outcomes in a rat model of transient focal cerebral ischemia.

## Materials and Methods

### Materials

Nylon thread was purchased from Beijing Sha Dong Biological Technology Co., Ltd. (Beijing, China), while 2,3,5-triphenyltetrazolium chloride (TTC) was purchased from Amersco (Solon, OH, United States). Antibodies were supplied by Protein Tech Group (Chicago, IL, United States). DAPI, 4% paraformaldehyde, phosphate buffered saline (PBS), polyvinylidene fluoride membrane (PVDF) membranes, 10% chloral hydrate, RIPA buffer, and bicinchoninic acid assay (BCA) kits were obtained from Beyotime Biotechnology (Beijing, China). All chemicals used in this study were of analytical reagent grade.

### Animals

The present study was approved by the Ethics Committee for Animal Experimentation of Guiyang College of TCM (Guizhou, China), in accordance with internationally recognized standards. Male Wistar rats (weight: 250–280 g) were obtained from Vital River Laboratory Animal Technology Co., Ltd. (Certificate Number: SCXK Jing 2012-0001). Rats were provided *ad libitum* access to standard rodent food and water and were housed under a 12-h light/dark cycle at 12 at a temperature of 22 ± 2°C.

### Transient Focal Cerebral Ischemia Model

A rodent model of transient focal cerebral ischemia was established via middle cerebral artery occlusion (MCAO) with an intraluminal suture for 1.5 h, followed by 24 h of reperfusion, as previously described (Wang et al., 2007). Briefly, rats were anesthetized via intraperitoneal injection of 10% chloral hydrate (400 mg/kg). Body temperature was maintained at 37 ± 0.5°C throughout the surgery using a heating blanket. The entire carotid artery (except the right side) was exposed via a 2.0 cm ventral midline neck incision, following which the three carotid arteries (left common/external/internal carotid arteries) were ligated. The origin of the MCA was blocked using 3–0 MCAO monofilament nylon thread. A laser Doppler system (PeriFlux System 5010, Perimed, Stockholm, Sweden) was used to monitor regional cerebral blood flow (rCBF). Rats in which rCBF did not drop below 20% of baseline levels following MCAO were excluded from analysis. Sham-operated control rats underwent a similar procedure without MCAO. Following 2 h of ischemia, the monofilament was gently removed, reperfusion was performed, and the incision was closed.

### Extraction of Total Flavonoids From *Caragana*

A mixture of crushed *Caragana* microphylla (50 g) and ether was boiled for 4 h at 40°C. *Caragana* microphylla without ether was then subjected to two water baths with 800 and 600 ml of 70% ether, respectively. The filtrate was then washed two times in 70% ether, collected at 60°C, and run through a resin column. The mixtures were decolored two times using distilled water and 70% ether, respectively. Finally, the total flavonoids (70.7 ± 0.4%) were obtained using UV-vis spectrophotometry.

### Animal Treatments

Following MCAO, rats were divided into the following five treatment groups: Group 1, sham operation (control); Group 2, MCAO treated with water (model); Group 3, MCAO treated with 15 mg/kg TFC per day (TFC 15 mg/kg); Group 4, MCAO treated with 30 mg/kg TFC per day (TFC 30 mg/kg); and Group 5, MCAO treated with 60 mg/kg TFC per day (TFC 60 mg/kg). Following 2 h of reperfusion, rats underwent intragastric administration of TFC for 14 days. An equal amount of saline was used in the negative control and model groups. On the 14th day, the rats were euthanized, and their brains were collected for measurements of mRNA and protein expression.

### Neurological Assessments and Measurement of Infarct Volume

Assessments of neurological function were performed following reperfusion, in accordance with previously described methods (Longa et al., 1989). Neurological function was assessed using the modified five-point scale scoring system (0–4), with higher scores indicative of more severe neurological impairment. Rats with scores of 1–3 points following MCAO were used for analysis. Model rats were sacrificed under anesthesia on the third day after surgery for TTC staining. The rat brains were cut into small sections (2.0 mm), immersed in 2% TTC, and fixed in 4% paraformaldehyde. Red staining indicates normal brain tissue, while pale gray areas represent infarcted tissue. Image-Pro Plus 6.0 software (Media Cybernetics) was used to calculate infarct volumes.

### Magnetic Resonance Imaging (MRI)

Rats were anesthetized throughout the MRI procedure using inhalation anesthetics [nitrous oxide/oxygen/isoflurane mixture (70%/30%/1.5%)]. Rats were placed in a suitable position, and their heads were fixed with a non-magnetic cradle. Infarct evolution was monitored using a 7 T Clinscan animal MRI system (Bruker, Ettlingen, Germany), which was used to acquire images at each imaging time point. All rats underwent MRI scanning at 14 days after MCAO, as previously described (Cai et al., 2016).

### Micro-Positron Emission Tomography (Micro-PET)

Micro-PET was performed 14 days after cerebral ischemia and reperfusion. In order to promote the uptake of fluorodeoxyglucose (FDG), which was allowed to circulate in the blood for 1 h, rats were fasted for at least 12 h prior to PET. Rats were positioned on the scanning bed following injection of FDG into the tail vein. An Inveon PET scanner (Siemens, Germany) was used to obtain the images and acquire regions of interest in the OSEM 3D format (Cai et al., 2016).

### Immunofluorescence Staining

Rat brains were fixed in 4% paraformaldehyde overnight at 4°C, following which they were cut into 5-μm using a microtome. The brain sections were then cultured with anti-CD31, anti-VEGF, anti-Ang-1, anti-HIF-1α, anti-Dll4, and anti-Notch1 (1:200) antibodies overnight at 4°C, followed by incubation with secondary antibodies for 1.5 h at 25°C. DAPI was used to stain the cell nuclei. Immunofluorescence images were obtained using an Olympus microscope equipped with a BX51 digital camera (Olympus, Japan) and quantified using IPP 6.0 software.

### Quantitative Polymerase Chain Reaction (qPCR)

Total RNA was collected and isolated 14 days after MCAO using Trizol reagent (Invitrogen, United States), purified using the RNeasy Micro Kit (Qiagen, Valencia, CA, United States) with a genomic DNA eliminator, and reverse transcribed to cDNA using a PrimeScript RT Reagent Kit (Takara, Dalian, China), as previously described (He et al., 2017). The qPCR procedure was performed using an ABI instrument (Applied Biosystems, United States) and fluorescent dye (SYBR Green I, Takara), in accordance with the following protocol: denaturation for 3 min at 94°C, followed by 40 cycles of 5 s at 94°C and 34 s at 60°C. The relative mRNA expression in each treatment group was normalized to levels of the housekeeper gene GAPDH and analyzed using the 2^-ΔΔCT^ method (He et al., 2017). Primer sequences are listed in **Table 1**.

**Table 1:** The primers sequences of selected genes designed for qPCR.

mRNA	Primer pairs
GAPDH	Forward: CTCTAAGGCTGTGGGCAAGGTCAT
	Reverse: GAGATCCACCACCCTGTTGCTGTA
CD31	Forward: TTTCGCTGCCAAGCTGGCGT
	Reverse: CCACCTGCACGCTGCACTTGAT
Ang-1	Forward: GTCACTGCACAAAAGGGACA
	Reverse: GGCTTACAAGGATGGCGTTA
VEGF	Forward: CGACAGAAGGGGAGCAGA AAG
	Reverse: GCACTCCAGGGCTTCATCATT

### Western Blotting

The ischemic ipsilateral cortices of the rat brains were quickly homogenized on ice, following which the samples were sonicated in RIPA buffer (Beyotime, China) containing phosphatase and protease inhibitor. The lysates were centrifuged, and the supernatant was collected. The concentrations of all proteins were detected using a bicinchoninic acid assay (BCA) Kit (Beyotime, China). Total proteins were separated via SDS-PAGE and transferred to PVDF membranes. The membranes containing proteins were blocked in 5% bovine serum albumin (BSA) at 25°C for 30 min; cultured with anti-VEGF, anti-Ang-1, anti-HIF-1α, anti-Dll4, and anti-Notch1and β-actin antibodies overnight at 4°C; and incubated with secondary antibodies for 1.5 h at 25°Con the following day. Chemiluminescence reagents were used to visualize the hybridization.

### Statistical Analysis

All data are represented as the mean ± the standard deviation (SD). All analyses were performed using SPSS version 22.0 (SPSS). Differences between two groups were evaluated using Student’s *t-test*. Differences among multiple groups were evaluated via one-way analyses of variance (ANOVA). The level of statistical significance was set at *P* < 0.05.

## Results

### Changes in rCBF

**FIGURE 2:**
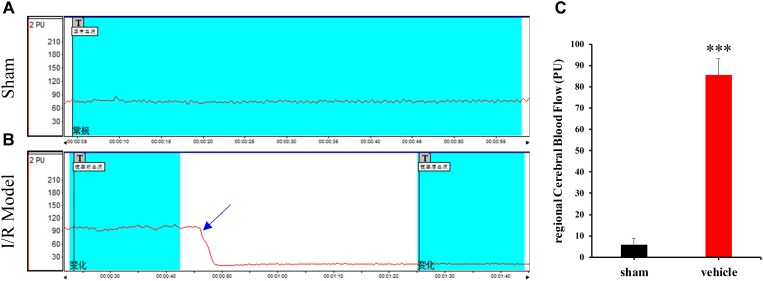
Changes in regional cerebral blood flow (rCBF) in the sham-operated **(A)** and model **(B)** groups. “T” in **(A,B)** indicates timing of different treatments. **(C)** The percentage of rCBF in the sham-operated and vehicle groups. Data are expressed as the mean ± standard deviation (SD). Statistical analysis: ^∗∗∗^*P* < 0.001, relative to the sham-operated group.

Laser Doppler flowmetry was used to evaluate changes in rCBF in sham-operated and MCAO rats. MCAO rats exhibited a 65–85% decrease in rCBF following removal of the occluding thread (**Figure 2B**). However, no variations were observed in the sham-operated group (**Figure 2A**). Thus, significant differences in rCBF were observed between sham-operated and MCAO rats (**Figure 2C**, ^∗∗∗^*p* < 0.0001). These findings indicate that rat models of cerebral ischemia-reperfusion injury were successfully established, although approximately 15% of rats in each group were excluded from data analysis due to failed surgery.

### Effects of TFC on Ischemic Brain Injury in Rats

**FIGURE 3:**
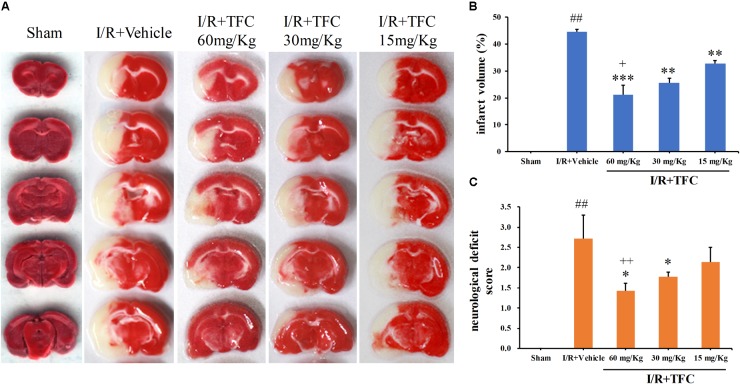
TFC decreased neurological deficit scores and cerebral infarct volume 3 days after MCAO. **(A)** Representative images of TTC-stained brain slices. The unstained area is defined as the infarct zone, while red staining indicates normal tissue. **(B)** Quantitative analysis of cerebral infarct volume according to TTC results. **(C)** Neurological deficit scores were evaluated after 2 h of ischemia and 22 h of reperfusion. Data are expressed as the mean ± standard deviation (SD) (*n* = 12). Statistical analysis: ^∗^*P* < 0.05, ^∗∗^*P* < 0.01, ^∗∗∗^*P* < 0.001; ^##^P < 0.01 vs. Sham group; ^+^P < 0.05, ^++^P < 0.01 vs. group treated with TFC (15 mg/kg), relative to the model group. TFC, total flavonoids in *Caragana*; MCAO, middle cerebral artery occlusion.

To investigate the influence of TFC on ischemic brain injury, MCAO rats were treated with different concentrations of TFC (15, 30, 60 mg/kg), following which we assessed neurological deficit scores and infarct volumes. As shown in **Figure 3C**, model-vehicle rats exhibited significantly poorer functional outcomes than sham-operated rats (^∗∗^*p* < 0.001). Nevertheless, the TFC treatment groups (15, 30, 60 mg/kg) exhibited significant, concentration-dependent improvements in functional outcomes following MCAO when compared with the model-vehicle group (^∗^*p* < 0.005). TFC at a test concentration of 60 mg/kg had the greatest effect on functional outcomes at 14 days after MCAO. The infarct areas were further analyzed via TTC staining, which indicated that TFC administration (15, 30, 60 mg/kg) markedly attenuated infarct volumes in the experimental groups when compared with those in the vehicle group at 3 days after MCAO (**Figure 3A**). Statistical analysis indicated that TFC treatment significantly decreased infarct volumes in a concentration-dependent manner (**Figure 3B**) (^∗∗^*p* < 0.001, ^∗∗∗^*p* < 0.0001). TFC treatment at the highest concentration (60 mg/kg) was associated with the greatest reductions in infarct volume. These results suggest that TFC exerts neuroprotective effects in an experimental model of ischemic stroke-induced brain injury.

### TFC Enhances Cerebral Perfusion in Ischemic Brain Tissue

**FIGURE 4:**
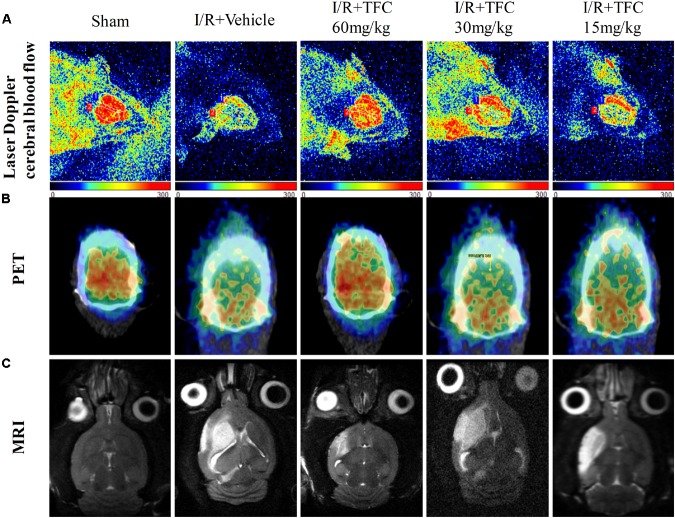
Exposure to different concentrations of TFC (60, 30, 15) significantly increased cerebral blood flow relative to that in the sham-operated group. **(A)** Representative laser Doppler images of cerebral blood flow (CBF). Orange and blue: low CBF; red: high CBF. **(B)** PET images of rat brains following treatment with different concentrations of TFC. Blue and green: low CBF; yellow and red: high CBF. **(C)** Brain MRI in rats treated with different concentrations of TFC. TFC, total flavonoids in *Caragana.*

To examine the effects of TFC on ischemic brain injury, three different methods (laser Doppler, micro-PET/CT, and MRI) were used to investigate cerebral blood flow. Representative laser Doppler images (**Figure 4A**) indicated that sham-operated rats exhibited normal cerebral blood flow, while model rats exhibited striking decreases in cerebral blood flow when compared with those in the sham-operated group. Treatment with TFC increased cerebral blood flow in a concentration-dependent manner. TFC at a concentration of 60 mg/kg exerted the most significant effect on cerebral blood flow.

PET with ^18^F-FDGis a powerful, non-invasive tool for evaluating the efficacy of cerebrovascular disease (Cross and Minoshima, 2013). Our results indicated that ^18^F-PDG uptake was notably higher in the TFC groups than in the vehicle group at 14 days after surgery (**Figure 4B**). These results indicate that TFC can effectively promote cerebral perfusion in the ischemic border region in a rat model of stroke.

To further detect the influence of TFC on ischemia-reperfusion injury, MRI was used to analyze infarct volumes. Infarct volume was significantly greater in the model group than in the sham-operated group (**Figure 4C**). However, infarct volumes were significantly lower in the TFC treatment than in the model group. Greater reductions in infarct area and volume were observed as the concentration of TFC increased. The strongest effects were observed at a TFC concentration of 60 mg/kg. Taken together, these results indicate that TFC exerts a protective effect by reducing infarct volume and enhancing cerebral blood flow following MCAO.

### TFC Promotes Angiogenesis Factor Expression in MCAO Rats

**FIGURE 5:**
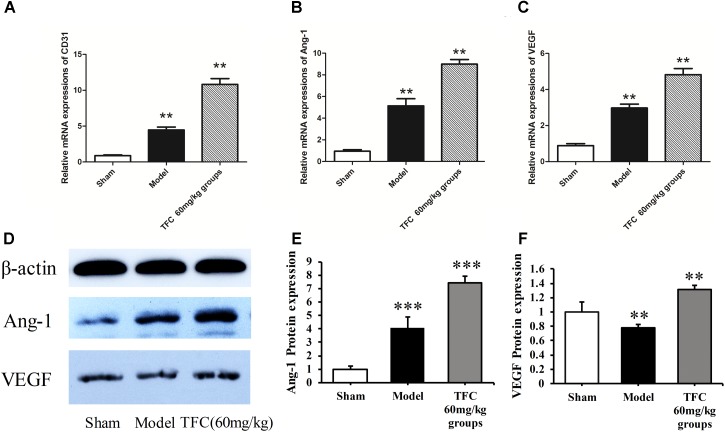
Influence of TFC (60 mg/kg) on the expression of CD31, Ang-1, and VEGF in cerebral homogenates 14 days after MCAO-reperfusion injury in rats. Quantitative PCR analysis of CD31 **(A)**, Ang-1 **(B)**, and VEGF expression **(C)**. Western blotting results for Ang-1, VEGF, and β-actin **(D)**. Quantitative results of Western blotting for Ang-1 **(E)** and VEGF **(F)** relative to β-actin. Data are presented as the mean ± standard deviation (SD) (*n* = 12). Statistical analysis: ^∗∗^*p* < 0.01 vs. Sham group, ^∗∗∗^*p* < 0.001. TFC, total flavonoids in *Caragana*; Ang-1, angiopoietin-1; VEGF, vascular endothelial growth factor.

**FIGURE 6:**
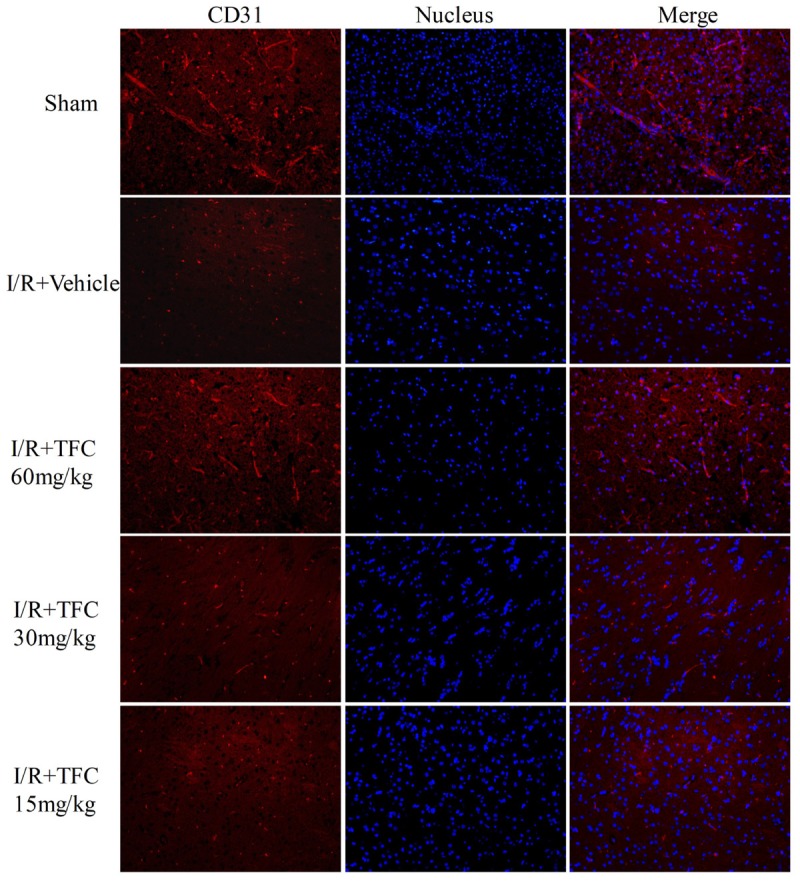
CD31 (endothelial cell marker) immunofluorescence staining of the ischemic area of the cortex in rats treated with different concentrations of TFC (60, 30, 15 mg/kg), sham rats, and vehicle rats. Scale bar = 25 μm. TFC, total flavonoids in *Caragana.*

**FIGURE 7:**
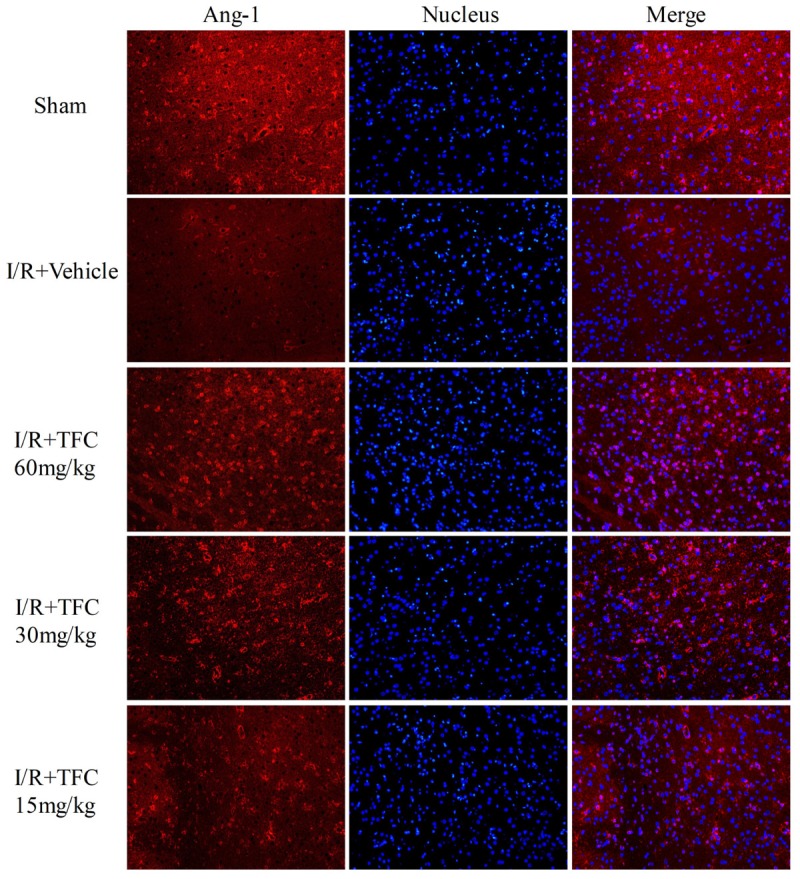
Immunofluorescence staining for Ang-1. Treatment with TFC (60, 30, 15 mg/kg) significantly increased the number of Ang-1-positive cells, relative to that in vehicle-treated rats. Scale bar = 25 μm. TFC, total flavonoids in *Caragana*; Ang-1, angiopoietin-1.

**FIGURE 8:**
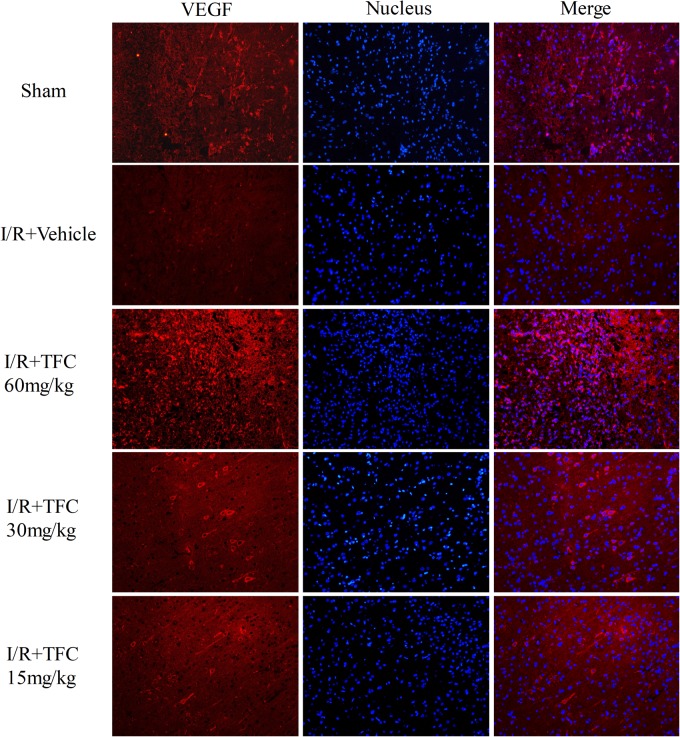
VEGF immunofluorescence staining was performed in each group after 14 days of TFC treatment at different concentrations (15, 30, 60 mg/kg), and in the sham and vehicle groups. Scale bar = 25 μm. VEGF, vascular endothelial growth factor.

**FIGURE 9:**
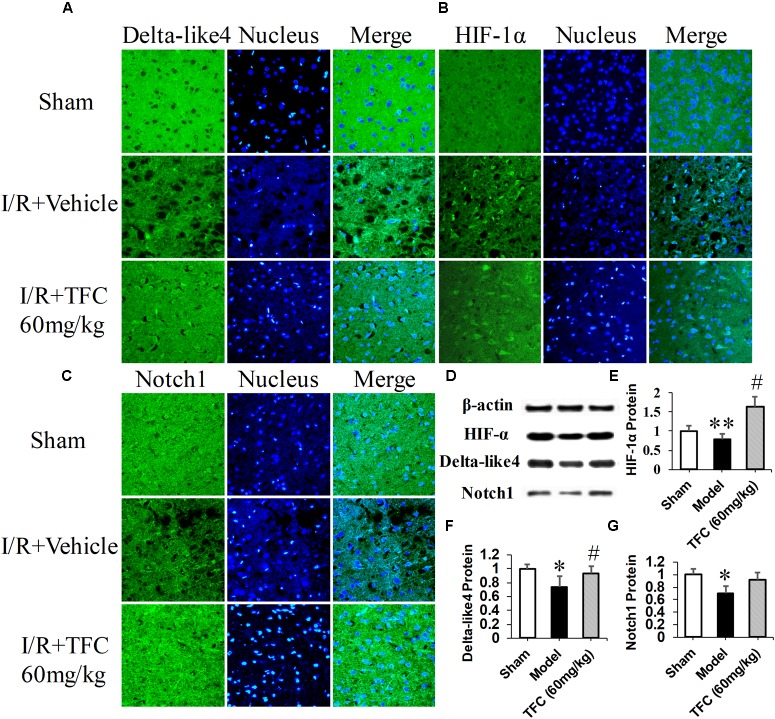
Delta-like 4 **(A)**, HIF-1α **(B)**, Notch1 **(C)** immunofluorescence staining were performed in sham rats, vehicle rats and TFC (60 mg/kg) treated rats. Scale bar = 25 μm. Western blotting results for Delta-like 4, HIF-1α, Notch1, and β-actin **(D)**. Quantitative results of Western blotting for Delta-like 4 **(E)**, HIF-1α **(F)**, and Notch1 **(G)** relative to β-actin. Data are presented as the mean ± standard deviation (SD) (*n* = 12). Statistical analysis: ^∗^*p* < 0.05, ^#^*p* < 0.05, ^∗∗^p < 0.01 vs. Sham group.

To investigate the mechanisms underlying the effects of TFC on cerebral ischemia, we examined CD31, Ang-1, and VEGF expression via q-PCR, Western blotting, and immunofluorescence analyses. As shown in **Figures 5A–C**, the expression of CD31, Ang-1, and VEGF mRNA was significantly increased in ischemic cortical tissue following surgery. Furthermore, TFC treatment significantly increased CD31, Ang-1, and VEGF mRNA expression in the experimental groups when compared with that in the vehicle group at 14 days after MCAO (^∗∗^*p* < 0.001). Similarly, Ang-1 and VEGF protein expression was also up-regulated following treatment with TFC (60 mg/kg) (**Figure 5D**). Statistical analysis of Western blotting results revealed significant differences between the model and treatment groups (^∗∗^*p* < 0.001, ^∗∗∗^*p* < 0.0001) (**Figures 5E,F**). Immunofluorescence analyses further demonstrated that TFC promoted the production of CD31, Ang-1, and VEGF in a concentration-dependent manner, with the greatest effects observed at a concentration of 60 mg/kg (**Figures 6–8**). In addition, the other three angiogenesis related factors: Dll4, HIF-1, and Notch1 were confirmed by Western blotting and immunofluorescence. The results of **Figures 9A–C** showed that TFC with the concentration of 60 mg/kg highly enhanced the production of Dll4, HIF-1, and Notch1 by immunofluorescence analysis. Meanwhile, it had been verified by Western blotting and its’ statistical analysis that the proteins expression of Dll4, HIF-1, and Notch1 were significantly up-regulated after treatment with TFC (60 mg/kg) (**Figures 9D–G**).

## Discussion

The present study is the first to demonstrate a significant and beneficial role of TFC in a rat model of transient focal cerebral ischemia. Our analysis revealed that TFC treatment at a concentration of 60 mg/kg can significantly improve neurological function and enhance cerebral perfusion when administered after transient MCAO in rats, similar to effects previously observed following treatment with puerarin (Lim et al., 2013; Wu et al., 2014). Our results further suggested that these effects are associated with up-regulation of the critical angiogenesis related factors CD31, VEGF, Ang-1, HIF-1α, Dll4, and Notch1 (Dvorak et al., 1995; Carmeliet and Collen, 1997; Thurston et al., 2000; Yancopoulos et al., 2000; Gale et al., 2004; Brindle et al., 2006; Hainaud et al., 2006; Al Sabti, 2007; Wang et al., 2007; Kume, 2012; Chen et al., 2014; Li et al., 2014; Meng et al., 2014; Yang et al., 2015; Doeppner et al., 2016; Shi et al., 2016, 2018).

The results of the present study establish the efficacy and dose–response relationship for the neuroprotective/angiogenic effects of TFC in rat model of transient MCAO. Relative to vehicle-treated rats, rats of the TFC treatment groups (15, 30, 60 mg/kg) exhibited significant, concentration-dependent reductions in infarct area and volume, improvements in neurological outcomes, and increases in cerebral blood flow following transient MCAO. The most remarkable effects were observed in rats treated with the highest dose of TFC (60 mg/kg). *Caragana sinica*, one of the most important herbs in traditional Chinese medicine, has been shown to improve a variety of symptoms associated with colds, strain-induced fatigue, and asthma. Additional studies have indicated that *Caragana sinica* promotes blood flow, nourishes the blood, clears lung-heat, promotes kidney, and spleen function, and aids in the healing of bruises/contusions (Jiangsu New Medical College, 1986; Jia et al., 1997). Flavonoids are the main active compounds in *Caragana sinica* and are known to exhibit cytoprotective, anti-inflammatory, anticancer, antioxidative, antiviral, and antibacterial properties (Wang et al., 2010, 2014; Al-Nakkash et al., 2012; Li et al., 2012; Lim et al., 2013; Lin et al., 2015; He et al., 2017). In the present study, we focused on the influence of the following four components: quercetin, 6,3′-dimethoxy-7,5′-dihydroxy isoflavone, caraphenol C, and (-)-ampelopsin F. Gao et al. (2009) reported that puerarin exerts neuroprotective effects in rats when administered intraperitoneally after transient MCAO, possibly due to increases in erythropoietin activity. In accordance with these findings, our results indicated that these components extracted from *Caragana sinica* exert significant neuroprotective/angiogenic effects.

We further examined the expression of CD31, VEGF, Ang-1, HIF-1α, Dll4, and Notch1 to investigate the mechanisms underlying the neuroprotective and angiogenic effects of TFC treatment in MCAO rats. TFC treatment increased VEGF mRNA and protein levels following reperfusion, thereby enhancing angiogenesis. Previous studies have demonstrated that VEGF plays a major role in tMCAO by inducing angiogenesis and neuroprotection (Mateos et al., 2016; Yousuf et al., 2016; Rezaee et al., 2018). Ang-1 promotes angiogenesis in physiological and pathological states during embryonic and postnatal life, and is essential for vascular maturation and stabilization (Carmeliet and Collen, 1997; Thurston et al., 2000; Brindle et al., 2006; Al Sabti, 2007). As Ang-1 may protect the peripheral vasculature from vascular leakage following ischemic injury, further studies are required to determine the role of Ang-1 in long-term neurological recovery after ischemic stroke. Our findings indicated that treatment with TFC at 60 mg/kg significantly up-regulated the expression of Ang-1 mRNA and protein in a rat model of transient MCAO. In mammals, HIF-1α is necessary for the early development of the brain and it upregulation induces neuroprotection and enhances angioneurogenesis (Li et al., 2014; Doeppner et al., 2016). VEGF signaling pathway can be regulated by the HIF-1α and Notch signaling pathway in the process of angiogenesis. Meanwhile, Dll4 is the ligand of the Notch1 and plays a paramount role in angiogenesis (Gale et al., 2004; Hainaud et al., 2006; Kume, 2012). In this study, we have confirmed that these three angiogenesis related factors were enhanced after treatment with TFC and showed neuroprotection and angioneurogenesis consistent with previous studies. In accordance with previous findings, we also observed increases in the expression of the endothelial cell marker CD31 in the TFC treatment groups (Musumeci et al., 2015). Taken together, our findings demonstrate that TFC enhances angiogenesis and improves neurological function in a rat model of MCAO mainly by up-regulating the expression of CD31, VEGF, Ang-1, HIF-1α, Dll4, and Notch1.

Epidemiological studies have found that morbidity of human stroke positive correlate to age, and aged brain does not respond exactly to ischemia compared to the young brain (Popa-Wagner et al., 2006; Buga et al., 2014; Sandu et al., 2016). Based on this we will establish MCAO model in aged rats to observe TFC neuroprotective effects and its mechanism in future study, which are closer to the clinic and more persuasive.

In summary, the results of the present study indicate that TFC (15, 30, 60 mg/kg) attenuates neurological deficits, reduces infarct volume, and promotes angiogenesis following MCAO in a concentration-dependent manner. In particular, when TFC was administered at 60 mg/kg, remarkable increases in the expression of CD31, VEGF, Ang-1, HIF-1α, Dll4, and Notch1 were observed following MCAO. Further studies are required to determine the clinical usefulness and potential mechanisms of TFC in patients with cerebral focal ischemic stroke.

## Author Contributions

QH, SL, and LL contributed equally to the conception and design of the study. NW was responsible for data collection. XF and SK processed and analyzed the data. QH and FH drafted the manuscript. All authors revised and approved the final draft of this article.

## Conflict of Interest Statement

The authors declare that the research was conducted in the absence of any commercial or financial relationships that could be construed as a potential conflict of interest.
